# Gene Mutation Annotation and Pedigree for Pulmonary Arterial Hypertension Patients in Han Chinese Patients

**DOI:** 10.5334/gh.1002

**Published:** 2021-10-18

**Authors:** Mei-Tzu Wang, Ming-Ji Charng, Pei-Ling Chi, Chin-Chang Cheng, Cheng Chung Hung, Wei-Chun Huang

**Affiliations:** 1 Department of Critical Care Medicine, Kaohsiung Veterans General Hospital, Kaohsiung, TW; 2 Section of Cardiology, Department of Internal Medicine, Kaohsiung Veterans General Hospital, Kaohsiung, TW; 3 School of Medicine, National Yang-Ming University, Taipei, TW; 4 Division of Cardiology, Department of Medicine, Taipei Veterans General Hospital, TW; 5 Department of Medical Education and Research, Kaohsiung Veterans General Hospital, Kaohsiung, TW; 6 Department of Pathology and Laboratory, Kaohsiung Veterans General Hospital, Kaohsiung, TW; 7 Department of Physical Therapy, Fooyin University, Kaohsiung, TW

**Keywords:** ClinVar database, DAVID database, gene annotation, gene mutation, pulmonary arterial hypertension, heritable pulmonary arterial hypertension

## Abstract

**Background:**

The etiology of pulmonary arterial hypertension (PAH) in the Han Chinese population is poorly understood.

**Objectives:**

The aim of this study was to assess gene variants and associated functional annotations for PAH in Han Chinese patients.

**Methods:**

This is an ethnicity-based multi-centre study. Blood samples were collected from 20 PAH patients who volunteered for the study, and genetic tests were performed. The DAVID database was used to functionally annotate the genes *BMPR2, ALK1, KCNK3, CAV1*, and *ENG*. Associated diseases, functional categories, gene ontology, and protein interactions were analysed using the Functional Annotation Tool in the DAVID database. GEO and ClinVar databases were also used for further comparison with gene mutations in our study.

**Results:**

PAH patient with gene mutations were female predominant except for a single male with a *BMPR2* mutation. Locus variants in our study included ‘G410DfsX1’ in BMPR2, ‘ex7 L300P,’ ‘ex4 S110PfsX40,’ and ‘ex7 E295Afs96X’ in *ALK1*, ‘c.-2C>A (IVS1–2 C>A)’ in *CAV1*, and ‘ex8 D366Q’ in ENG were not found in the ClinVar database associated with PAH. In addition to BMP and TGF-β pathways, gene ontology of input genes in the DAVID database also included pathways associated with nitric oxide signaling and regulation.

**Conclusions:**

This Multi-centre study indicated that ‘G410DfsX1’ in BMPR2, ‘ex7 L300P,’ ‘ex4 S110PfsX40,’ ‘ex7 E295Afs96X’ in *ALK1*, ‘c.-2C>A (IVS1–2 C>A)’ in *CAV1*, and ‘ex8 D366Q’ in ENG were identified in Han Chinese patients with PAH. Females were more susceptible to PAH, and a relatively young age distribution was observed for patients with *BMPR2* mutations.

## Introduction

Pulmonary arterial hypertension (PAH) is defined by the presence of pre-capillary pulmonary hypertension, with right heart catheterisation showing mean pulmonary arterial pressure ≥20 mmHg, pulmonary artery wedge pressure ≤15 mmHg and a pulmonary vascular resistance >3 Wood units [1,2]. The National Organization for Rare Disorders (NORD) classified pulmonary hypertension into three subtypes, including idiopathic pulmonary arterial hypertension (IPAH), heritable pulmonary hypertension (HPH), and associated pulmonary hypertension [3]. Patients with a family history of PAH were grouped under the term ‘heritable PAH (HPAH)’ in group 1 PAH. This is an autosomal-dominant vascular disorder that predominantly affects pulmonary arterioles [4,5]. Furthermore, genetic mutations have been identified in sporadic primary PAH [6]. Some gene variants were considered to have potential effects on individual susceptibility to pulmonary hypertension for Chinese Han Chinese [3], suggesting that the variants of the PAH gene may be associated with the development of PAH.

Bone morphogenetic protein receptor type II (*BMPR2*) gene mutation is the single most common causal factor for HPAH, however, approximately 25% of idiopathic PAH patients have pathogenic mutations without prior family history of disease [7,8]. Previous large survey confirmed that *BMPR2* (15.3%), *ACVRL1* (activin receptor-like kinase 1 (*ALK1*)) (0.9%), *ENG* (endoglin) (0.6%), and *KCNK3* (potassium channel subfamily K member 3) (0.4%) are causal mutations of PAH [9]. *CAV1* (caveolin-1) functions to physically co-localize BMP receptors, and is associated with both lipodystrophy and PAH [10,11]. Although the mutated gene has been identified in white or Hispanic patients, these mutations in Han Chinese patients with PAH remain to be elucidated. The aim of this study is to analyse the gene variants and their associated functional annotations in Han Chinese PAH patients.

## Methods

### Data source and study population

This is a multi-centre ethnicity-based study that investigates PAH gene mutations in the Han Chinese. Twenty PAH patients were enrolled into this study. Informed consent was obtained from all participants and their family members for collection of blood samples and genetic analysis. The Institutional Review Board (IRB) of Kaohsiung Veterans General Hospital approved this study (IRB number: KSVGH21-CT1-21).

### Whole exome sequencing, alignment, variant calling and annotation

Genomic DNA was isolated from peripheral blood leukocytes of PAH patients. Polymerase chain reaction (PCR) was used to amplify the exons and flanking intronic bases of the five genes, including BMPR2 (13 exons), *ALK-1* (10 exons), *CAV1* (3 exons), *ENG* (14 exons), and *KCNK3* (2 exons). The primers used for PCR were designed using reference sequences deposited in the GenBank database. Standard DNA sequencing reactions were performed using the fluorescence-labelled dideoxy chain termination method with the BigDye Terminator ABI Prism Kit and the ABI PRISM™ 3700 DNA Analyser (Applied Biosystems, Foster City, CA, USA), according to the manufacturer’s instructions.

### Gene mutation and expression analysis

The DAVID database (https://david.ncifcrf.gov) was used to functionally annotate mutated genes of PAH patients in this study [12,13]. *BMPR2, ALK1*, KCNK3, *CAV1*, and *ENG* were entered into the gene list, with ‘Official_Gene_Symbol’ as the selected identifier, and human/homo sapiens as the selected species; background population was set as homo sapiens. Associated diseases, functional categories, gene ontology, and protein interaction were analysed using the Functional Annotation Tool in the DAVID database. The aforementioned genes were also entered into the GEO profiles database, where search results on mutations and expressions related to PAH were manually reviewed. Pulmonary hypertension was also used as a key word for the ClinVar database [14]; genetic locus mutations were downloaded from the website in text format, which were transformed to Excel format for further comparisons with gene mutations in our study.

### Statistical analyses

The SPSS version 22 (IBM, Chicago, IL, USA) was used for data analysis. Percentile values were used to express categorical data, and were analysed using the chi-square test. Mean (μ) and standard deviation values were used for continuous variables using the Student’s unpaired *t-tests*. A p-value of < 0.05 (–Log P value > 1.3) was considered to be statistically significant.

## Results

### Patient characteristics

All patients’ basic characteristics are listed in Table 1. There was one variant at ‘G410DfsX1’ in *BMPR2*, as well as three locus variants in *ALK1*, at ‘ex7 L300P,’ ‘ex4 S110PfsX40,’ and ‘ex7 E295Afs96X.’ In addition, one variant at ‘c.-2C>A (IVS1–2 C>A)’ in *CAV1*, and one at ‘ex8 D366Q’ in *ENG* were also found. There were 11 patients without gene mutations, whereas patient 15 (P15) had gene mutations at both ex7 L300P in *ALK1* and D366Q in *ENG*. Table 2 shows the comparison between gene subgroups, including gender, age, body height, and body weight. Out of the 20 patients, there were three (15%) males and 17 (85%) females, with a mean age of 48.6 ± 11.1. There was a 25-year-old male in the *BMPR2* group, who was considerably younger than patients in other groups.

**Table 1:** Basic characteristics and gene mutations for pulmonary artery hypertension patients.

Number	Sex	Age	BMPR2	ALK-1	KCNK3	CAV1	ENG

1	M	54	(－)	(－)	(－)	(－)	(－)
2	F	67	(－)	(－)	(－)	(－)	(－)
3	F	55	(－)	(－)	(－)	ex3 A216P	(－)
4	F	37	(－)	(－)	(－)	(－)	(－)
5	F	48	(－)	(－)	(－)	(－)	(－)
6	F	49	(－)	(－)	(－)	(－)	(－)
7	F	39	(－)	(－)	(－)	(－)	(－)
8	F	36	(－)	(－)	(－)	(－)	(－)
9	F	45	(－)	(－)	(－)	(－)	(－)
10	F	71	(－)	(－)	(－)	(－)	(－)
11	F	43	(－)	(－)	c.-2C>A (IVS1-2 C>A)	(－)	(－)
12	M	48	(－)	(－)	(－)	(－)	ex8 D366Q
13	M	25	G410DfsX1	(－)	(－)	(－)	(－)
14	F	57	(－)	(－)	(－)	(－)	(－)
15	F	48	(－)	ex7 L300P	(－)	(－)	ex8 D366Q
16	F	62	(－)	ex4 S110PfsX40	(－)	(－)	(－)
17	F	56	(－)	(－)	(－)	(－)	(－)
18	F	42	(－)	ex7 E295Afs96X	(－)	(－)	(－)
19	F	51	(－)	(－)	(－)	(－)	(－)
20	F	39	(－)	(－)	(－)	(－)	ex8 D366Q

**Table 2:** Comparison of variables between five gene subgroups.

Variables	All	BMPR2			ALK1			KCNK3			CAV1			ENG		

		(+)	(–)	P value	(+)	(–)	P value	(+)	(–)	P value	(+)	(–)	P value	(+)	(–)	P value

Count	20	1 (5%)	19 (95%)		3	17		1	19		1	19		3	17	
Male	3 (15%)	1 (100%)	2 (10.5%)	0.1500	–	3 (17.7%)	1.0000	–	3 (15.8%)	1.0000	–	3 (15.8%)	1.0000	1 (33.3%)	2 (11.8%)	0.4035
Female	17 (85%)	–	17 (89.5%)	–	3 (100%)	14 (82.4%)		1 (100%)	16 (84.2%)	–	1 (100%)	16 (84.2%)	–	2 (66.7%)	15 (88.2%)	
Age	48.6 ± 11.1	25	49.8 ± 9.9	–	50.7 ± 10.3	48.2 ± 11.5	0.7368	43.0	48.9 ± 11.3	–	55.0	48.3 ± 11.3	–	45.0 ± 5.2	49.2 ± 11.9	0.5570
Height	159.2 ± 6.4	161	159.1 ± 6.6	–	156.0 ± 3.6	159.7 ± 6.7	0.3683	154.1	159.9 ± 6.5	–	155.0	159.4 ± 6.5	–	163.3 ± 7.1	158.4 ± 6.2	0.2292
Weight	58.8 ± 9.3	62	58.6 ± 9.5	–	49.7 ± 1.2	60.4 ± 9.2	0.0633	55.6	59.0 ± 9.5	–	64.0	58.5 ± 9.5	–	62.8 ± 14.8	58.1 ± 8.5	0.4315

Values are expressed as mean (μ) ± standard deviation for continuous variables and numbers (%) for categorical variables.

### DAVID database analysis

**Figure 1:**
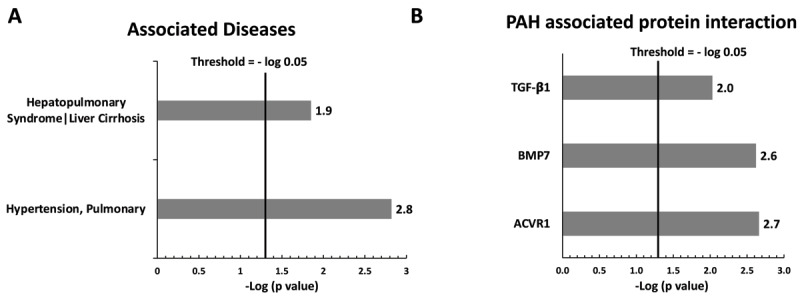
The DAVID database was used for functional annotation. Panel A. Mutated genes in this study, including *BMPR2, ALK1, KCNK3, CAV1*, and *ENG* were entered into the gene list. Common associated diseases included pulmonary hypertension, liver cirrhosis, as well as associated hepatopulmonary syndrome. Panel B. Mutated genes in our study and their associated protein interactions. Activin A receptor type 1 (ACVR1); bone morphogenetic protein 7 (BMP7); transforming growth factor beta receptor 1 (TGF-β1).

The DAVID database was used for functional annotation, and the output data are shown in Figure 1. Mutated genes in this study, including *BMPR2, ALK1*, KCNK3, *CAV1*, and *ENG*, are displayed in the gene list. Commonly associated diseases for these mutations included pulmonary hypertension (-Log P value: 1.9), liver cirrhosis, and associated hepatopulmonary syndrome (–Log P value: 2.8) (Fig. 1A). Mutated genes and their associated proteins included transforming growth factor beta receptor 1 (*TGF-β1*) (–Log P value: 2.0), bone morphogenetic protein 7 (*BMP7*) (Log P value: 2.6), and activin A receptor type 1 (*ACVR1*) (–Log P value: 2.7), as shown in Figure 1B.

**Figure 2:**
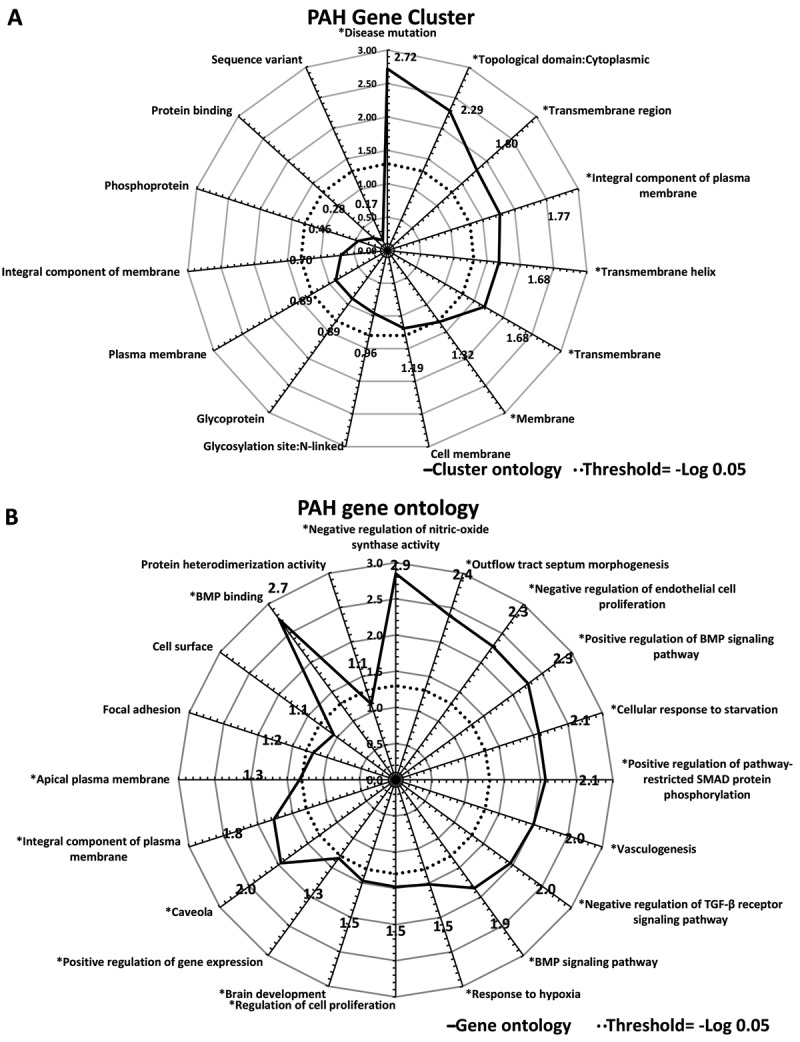
Gene cluster and ontology outputs of the five locus variants, as analysed by the DAVID database. Panel A. All mutated genes in this study belonged to the ‘disease mutation’ gene cluster (-log P value: 2.72). Panel B. PAH gene ontology of mutated genes in this study. Gene ontology reports ‘negative regulation of nitric-oxide synthase activity’ (–log P value: 2.85), ‘negative regulation of endothelial cell proliferation’ (–log P value: 2.28), ‘positive regulation of BMP signalling pathway’ (–log P value: 2.26), ‘positive regulation of pathway-restricted Smad protein phosphorylation’ (–log P value: 2.07), ‘vasculogenesis’ (–log P value: 2.00), ‘negative regulation of TGF-β receptor signalling pathway’ (–log P value: 1.96), ‘regulation of cell proliferation’ (–log P value: 1.48), and ‘BMP binding’ (–log P value: 2.74). Bone morphogenetic protein (BMP); transforming growth factor beta receptor 1 (TGF-β1).

Gene cluster and ontologies were analysed, and the results are shown in Figure 2. All mutated genes in this study were found in the gene cluster of ‘disease mutation’ (–Log P value: 2.72) (Figure 2A). Moreover, gene ontologies of input genes included ‘negative regulation of nitric-oxide synthase activity’ (–Log P value: 2.85), ‘negative regulation of endothelial cell proliferation’ (–Log P value: 2.28), ‘positive regulation of BMP signalling pathway’ (–Log P value: 2.26), ‘positive regulation of pathway-restricted Smad protein phosphorylation’ (–Log P value: 2.07), ‘vasculogenesis’ (–Log P value: 2.00), ‘negative regulation of TGF-β receptor signalling pathway’ (–Log P value: 1.96), ‘regulation of cell proliferation’ (–Log P value: 1.48), and ‘BMP binding’ (–Log P value: 2.74), as shown in Figure 2B.

### GEO profile database analysis

**Figure 3:**
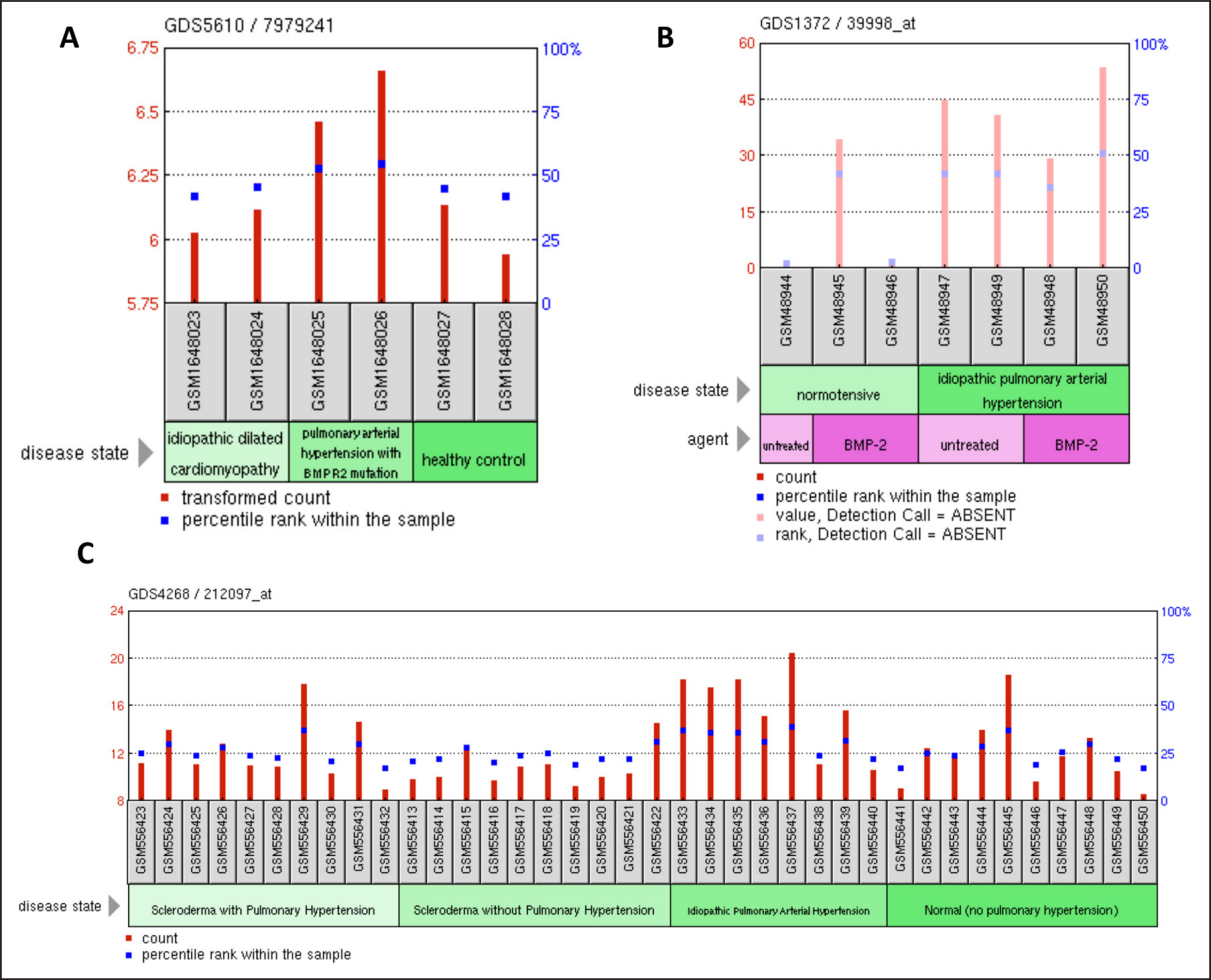
GEO profile database associated with gene mutations and expressions. Panel A. GEO profile database reports that *BMPR2* mutation is associated with PAH expression. Panel B. *BMP2* (39998_at) expression is associated with idiopathic pulmonary artery hypertension (IPAH). Panel C. *CVA1* (212097_at) expression could lead to IPAH. Abbreviations: Bone morphogenetic protein receptor (BMPR).

GEO profile database associated with gene mutations and expressions was shown in Figure 3. The data discussed in Figure 3A were deposited into the NCBI’s Gene Expression Omnibus (Edgar et al., 2002), which are accessible through GEO Series accession numbers. *BMPR2* mutation (GDS5610) is associated with PAH expression (Figure 3A) (https://www.ncbi.nlm.nih.gov/geo/query/acc.cgi?acc=GSE67492). BMP2 (GDS5610/39998_at) expression is associated with IPAH (Figure 3B) (https://www.ncbi.nlm.nih.gov/geo/query/acc.cgi?acc=GSE2559), and changes in CVA1 (212097_at) expression could lead to IPAH (Figure 3C) (https://www.ncbi.nlm.nih.gov/geo/query/acc.cgi?acc=GSE22356).

### Locations of gene mutations and family pedigrees

**Figure 4:**
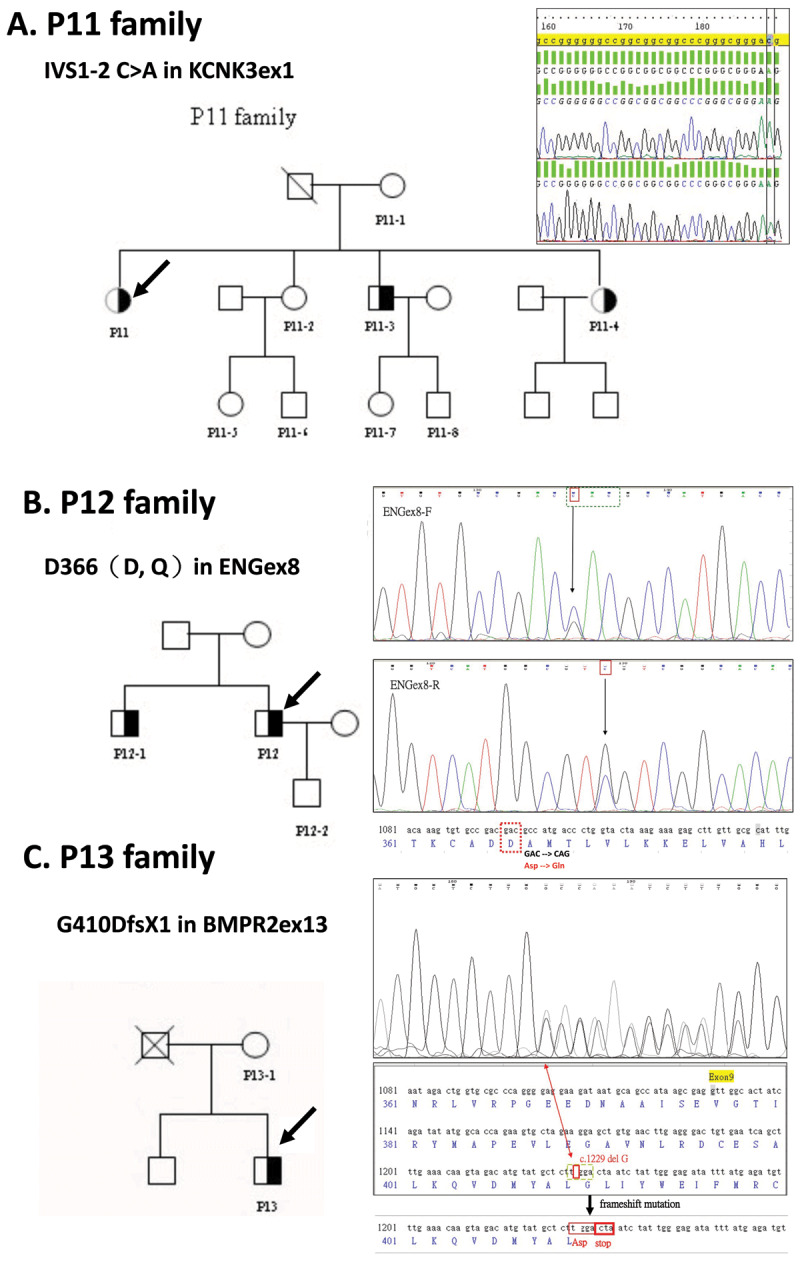
Location of gene mutation and family pedigree of volunteer PAH patients. Arrows indicate PAH patients in our study. Panel A. Gene mutation location of P11 is at IVS1–2 C>A in the KCNK3ex1 fragment. Panel B. P12 has a gene mutation located at D366(D, Q) in the ENGex8 fragment. Panel C. P13 has s gene mutation located at G410DfsX1 in the BMPR2ex13 fragment.

The locations of gene mutations and further details are illustrated in Table 3, and family pedigrees of volunteer PAH patients are shown in Figure 4. For example, gene mutation location of patient 11 (P11) was at IVS1–2 C>A in the KCNK3ex1 fragment (Figure 4A); patient 12 (P12) had a gene mutation located at D366 (D, Q) in the ENGex8 fragment (Figure 4B); patient 13 (P13) showed mutation at G410DfsX1 in the BMPR2ex9 fragment (Figure 4C).

**Table 3:** Mutation positions for PAH patients receiving pedigree survey.

Sample ID	Fragment	Gene Position	RefSeq Base Call	Base Call	Amino Acid Change	DNA concentration (ng/μl)

P11	KCNK3ex1	IVS1-2	C	A	IVS1-2 C>A	136
P12	ENGex8	1096	G	C	D366(D,Q)	113
P13	BMPR2ex9	1229	G	del G	G410DfsX1	106

## Discussion

This is a multi-centre ethnicity-based study for PAH gene mutations. Five new locus variants were reported in this study: one at ‘G410DfsX1’ in *BMPR2*, three in *ALK1*, at ‘ex7 L300P,’ ‘ex4 S110PfsX40,’ and ‘ex7 E295Afs96X.’ Moreover, one mutation was found at ‘c.-2C>A (IVS1–2 C>A)’ in *CAV1*, and one was found at ‘ex8 D366Q’ in *ENG*. Most of the patients in this study were female, except for one young male in the *BMPR2* group. The genes investigated are associated with common PAH-associated diseases, including pulmonary hypertension, liver cirrhosis, and associated hepatopulmonary syndrome. Mutated genes were involved in *TGF-β, BMP7*, and *ACVR1* interactions.

### Gene mutation for PAH was associated with age and gender difference

Compared with PAH patients without *BMPR2* mutations, those with *BMPR2* mutations were younger, with a mean age of 35.4 years [8]. Previous study indicated a mean age of 42 years for *BMPR2* non-carriers [8]. Patients with gene mutations other than BMPR2 in our study were older than previously reported (49.7–62.8 years in *ALK1, KCNK3, CAV1*, and *ENG* subgroups). Our study also indicated that patients with *BMPR2* mutations were younger than patients with other gene mutations.

The occurrence of *BMPR2* mutations in sporadic PAH cases without a family history can be attributed to low penetrance of *BMPR2* mutations (20%–30%) and *de novo* mutations [15]. The estimated penetrance in male and female carriers is 14% and 42%, respectively [15,16]. It has been shown that female is the single most important determinant for the penetrance of *BMPR2* mutations in PAH [15,16]; male patients were significantly more likely to have *BMPR2* mutations than female patients [17]. Similarly, in our study, only one male was found to have the *BMPR2* mutation.

PAH occurs predominantly in females; the sex ratio of female-to-male is 2.4:1. It has been suggested that oestrogen and its metabolism may be associated with the pathogenesis of PAH [16,18,19]. However, mortality rate of PAH is higher in males as compared with that of females, particularly in male *BMPR2* mutation carriers [20]. The Registry to Evaluate Early and Long-Term Pulmonary Arterial Hypertension Disease Management (REVEAL) showed a higher female predominance of PAH irrespective of *BMPR2* status, and found that the female-to-male ratio for PAH was 3.9:1 in races other than whites, black and Hispanic [21]. Our study results agreed with previous evidence that suggested female dominance in IPAH or HPAH (Table 1) [17].

### Gene ontologies from the Han Chinese patients correlated with PAH mechanism

Mutated genes in our study, including *BMPR2, ALK1*, KCNK3, *CVA1*, and *ENG* were considered to have evidence of mutation in patients with PAH [22]. Among which, *BMPR2, ALK1* and *ENG* were clearly recognized for their biological functions in PAH [22]. *BMPR2* is particularly highly expressed on the cell surface of pulmonary vascular endothelium [23], and *BMP9* functions as a circulating vascular quiescence factor to counterbalance cell apoptosis and excessive proliferation in endothelial cells [24]. In *BMPR2* mutated pulmonary artery smooth muscle cells, there was a loss of antiproliferative effects from BMPs, resulting in excessive Smad1/5 signalling, which led to hyper-proliferative cells [24]. The *BMPR2* protein forms a complex with *ALK1; ENG* plays a co-receptor to form a complex on the membrane and signal specifically in response to the circulating BMP ligands [25]. *CAV1* is highly expressed in endothelial cells, and is an important constituent protein of caveolae. BMP receptors are localized in caveolae, and loss of *CAV1* inhibits *BMPR*2 membrane localization and signalling [26,27]. *KCNK3* encodes a potassium channel that generates the membrane potential needed to regulate pulmonary vascular tone [28]. *BMPR2*, composed of an extracellular motif and transmembrane kinase domains, and is a part of the TGF-β receptor superfamily [29]; mutated genes associated with the TGF-β signalling pathway include *ALK1*, CVA1, and ENG [30]. All of the genes analyzed in our study encode for membrane or transmembrane proteins [22].

The TGF-β pathway processes angiogenesis via two distinct signalling pathways, including the ALK5-Smad2/3 pathway and the ALK1-Smad1/5/8 pathway [31]. Endoglin counterbalances the stabilizing role of ALK5 to stabilise the vessel and inhibit endothelial cell overproliferation [32]. However, *BMPR2* or *ACVRL1* mutation disrupt the SMAD1/5/8 pathway and BMP signalling. This inhibits SMC apoptosis, which leads to SMC proliferation, vascular remodelling, and ultimately results in PAH [33]. Our study (Figure 2) reported gene ontologies, including ‘positive regulation if BMP signaling pathway’ and ‘negative regulation of TGF-β signaling pathway,’ which confirmed the imbalance between the ALK5-Smad2/3 pathway and ALK1-Smad1/5/8 pathways in in these gene mutations, resulting in PAH. Gene ontology of input genes in our study also suggested that in addition to their roles in BMP and TGF-β pathways, they also function in pathways associated with nitric oxide signalling and regulation, which coincides with the pathobiology of PAH; the results of this study was supported by a previous study [22].

### New pathogenic mutations for IPAH might exist in the Han Chinese population

There are differences in genetic, physiological, and anatomic factors between races, which affected the structure and function of the right ventricle [34]. However, studies focused on the Han Chinese are limited. The REVEAL trial enrolled only 3.3% Asians, and it showed a higher PAH prevalence in Hispanic patients as compared with that of earlier registries [21]. A Chinese registry reported a lower 1-year survival rate for Chinese PAH patients [35]. Early molecular genetics studies can strengthen clinical diagnosis and assist decision-making in adopting effective treatment [36].

Locus variants in our study included ‘G410DfsX1’ in *BMPR2*, ‘ex7 L300P,’ ‘ex4 S110PfsX40,’ and ‘ex7 E295Afs96X’ in *ALK1*, ‘c.-2C>A (IVS1–2 C>A)’ in *CAV1*, and ‘ex8 D366Q’ in *ENG*, which were not found in the ClinVar database associated with PAH. Further studies are needed to determine whether these new mutations are associated with sporadic primary PAH or HAPH in the Han Chinese. This study also revealed similar gender and age distribution with previous large studies, which implies that other genetic mutations or environmental factors may contribute to the poor survival of Han Chinese PAH patients [35]. Further studies on offspring of these PAH patients may be needed to confirm the association between these new mutations in Han Chinese patients with PAH.

### Study limitations

The sample size of this study was small, and not all family members of enrolled PAH patients received whole genome sequencing. Although the characteristics of patients included in this study were similar to those of previous large studies. Further investigations are needed for PAH patients from the Han Chinese population.

### Study strengths

This is a multi-centre ethnicity-based study to determine gene mutations in PAH patients. Current trials are focused mostly on white or Hispanic patients; studies of PAH patients in the Han Chinese population are rare. This study focused on the Han Chinese and used databases, including DAVID, ClinVar, and GEO profiles to analyse the relationship between mutated genes and their functional ontologies. This study offers valuable genetic data for the Han Chinese population.

## Conclusions

This multi-centre ethnicity-based analysis revealed five new locus variants that is potentially associated with PAH in the Han Chinese, including ‘G410DfsX1’ in *BMPR2*, ex7 L300P,’ ‘ex4 S110PfsX40,’ and ‘ex7 E295Afs96X’ in *ALK1*, ‘c.-2C>A (IVS1–2 C>A)’ in *CAV1*, and ‘ex8 D366Q’ in *ENG*. This study reports that females have greater susceptibility to PAH in the Han Chinese; moreover, in addition to BMP and TGF-β pathways, changes in nitric oxide signalling and regulation have also been reported to be associated with PAH. This study further enriched the gene database for PAH in the Han Chinese, which may be used to advance PAH therapy at the molecular genetic level.

## Data Accessibility Statement

The data underlying this article cannot be shared publicly due to legal restrictions imposed by the government of Taiwan in relation to the ‘Personal Information Protection Act.’
